# In Vitro Anti-Oxidant, In Vivo Anti-Hyperglycemic, and Untargeted Metabolomics-Aided-In Silico Screening of Macroalgae Lipophilic Extracts for Anti-Diabetes Mellitus and Anti-COVID-19 Potential Metabolites

**DOI:** 10.3390/metabo13121177

**Published:** 2023-11-27

**Authors:** Anggit Listyacahyani Sunarwidhi, Wahyu Rahmaniar, Eka Sunarwidhi Prasedya, Hasriaton Padmi, Sri Widyastuti, Kukuh Waseso Jati Pangestu, Bq Tri Khairina Ilhami, Ervina Handayani, Ni Wayan Putri Utami, Farreh Alan Maulana, Muhammad Syach Maulad Ichfa, Ari Hernawan

**Affiliations:** 1 Department of Pharmacy, Faculty of Medicine, University of Mataram, Mataram 83115, Indonesia; 2 Bioscience and Biotechnology Research Centre, University of Mataram, Mataram 83115, Indonesia; 3 Institute of Innovative Research, Tokyo Institute of Technology, Yokohama 226-8503, Japan; 4 Department of Biology, Faculty of Mathematics and Natural Sciences, University of Mataram, Mataram 83115, Indonesia; 5 Faculty of Food Technology and Agroindustry, University of Mataram, Mataram 83115, Indonesia; 6 Department of Informatics Engineering, Faculty of Engineering, University of Mataram, Mataram 83115, Indonesia

**Keywords:** diabetes mellitus, COVID-19, macroalgae, lipophilic compounds, metabolomics-aided in silico

## Abstract

COVID-19 patients with comorbid DM face more severe outcomes, indicating that hyperglycemic conditions exacerbate SARS-CoV-2 infection. Negative side effects from existing hyperglycemia treatments have urged the need for safer compounds. Therefore, sourcing potential compounds from marine resources becomes a new potential approach. Algal lipids are known to possess beneficial activities for human health. However, due to limitations in analyzing large amounts of potential anti-hyperglycemic and anti-COVID-19-related marine metabolites, there is an increasing need for new approaches to reduce risks and costs. Therefore, the main aim of this study was to identify potential compounds in macroalgae *Sargassum cristaefolium*, *Tricleocarpa cylindrica*, and *Ulva lactuca* lipophilic extracts for treating DM and COVID-19 by an integrated approach utilizing in vitro anti-oxidant, in vivo anti-hyperglycemic, and metabolomic-integrated in silico approaches. Among them, *S. cristaefolium* and *T. cylindrica* showed potential anti-hyperglycemic activity, with *S. cristaefolium* showing the highest anti-oxidant activity. A GC-MS-based untargeted metabolomic analysis was used to profile the lipophilic compounds in the extracts followed by an in silico molecular docking analysis to examine the binding affinity of the compounds to anti-DM and anti-COVID-19 targets, e.g., α-amylase, α-glucosidase, ACE2, and TMPRSS2. Notably, this study reveals for the first time that steroid-derived compounds in the macroalgae *T. cylindrica* had higher binding activity than known ligands for all the targets mentioned. Studies on drug likeliness indicate that these compounds possess favorable drug properties. These findings suggest the potential for these compounds to be further developed to treat COVID-19 patients with comorbid DM. The information in this study would be a basis for further in vitro and in vivo analysis. It would also be useful for the development of these candidate compounds into drug formulations.

## 1. Introduction

Diabetes mellitus (DM) is still a global health problem. Individuals with DM are also prone to experience worse conditions when infected by other diseases, such as COVID-19 [1,2,3]. Increased disease severity and mortality among COVID-19 patients when DM co-occurs was observed. Patients suffering from DM experience more severe COVID-19 symptoms than non-diabetic patients [4,5,6], because DM is a risk factor for upregulated angiotensin-converting enzyme-2 (ACE2) gene expression due to hyperglycemic conditions [7,8]. Hyperglycemia in DM triggers glycosylation of the host ACE2 and viral S-protein [9], thereby increasing the binding between the virus and ACE2. This, in turn, facilitates the entry of the virus [10], thereby worsening the condition of COVID-19 patients [11]. Another study highlighted that COVID-19 patients with DM had increased levels of interleukin-6 and C-reactive protein compared with non-DM patients [12]. Some anti-DM drugs, such as α-glucosidase inhibitors, have been used against COVID-19 [13], and findings suggest that these compounds show neutral mortality rates in COVID-19 patients compared with other anti-DM drugs [14]. However, conventional treatments for DM have drawbacks, including gastrointestinal side effects, such as abdominal pain and diarrhea [15,16]. Further complications, including weight gain, cardiac conditions, anemia, headache, hypoglycemia, nausea, vomiting, fatigue, constipation, and dyspepsia, have also been associated with standard anti-DM drugs [17]. This highlights the urgent need for new compounds that can effectively combat hyperglycemia and COVID-19 but have fewer side effects. 

The search for novel drug compounds is increasingly turning to natural resources, with marine biota of particular interest. Macroalgae, abundant in tropical regions, have become a focal point in these efforts. Among them, *Sargassum cristaefolium*, *Tricleocarpa cylindrica*, and *Ulva lactuca* are the prominent macroalgae species found along the coast of Lombok in Indonesia. *S. cristaefolium* is a member of Phaeophyta (brown macroalgae), *T. cylindrica* is a member of Rhodophyta (red macroalgae), and *U. lactuca* is a member of Chlorophyta (green macroalgae). Previous studies have demonstrated the diverse pharmacological potential of macroalgae, including anti-oxidant, anti-aging, and anti-melanoma activities [18,19]. The lipophilic extract of the brown macroalga *Sargassum polycystum* was able to decrease hyperglycemia in alloxan-induced rats [20]. Another study also showed that macroalgae lipophilic compounds have the ability to reduce inflammation and oxidation [21,22,23]. Meanwhile, the *Ulva fasciata* lipid extract mainly containing palmitic acid has been shown to have an anti-oxidant and anti-genotoxic effect [24]. Moreover, non-flavonoid compounds from brown macroalgae show higher bioactivities than the other classes [25]. These non-flavonoid compounds include lipophilic substances, such as fatty acids, steroids, and terpenoids [25]. 

Fatty acids have demonstrated efficacy against metabolic diseases, including DM [26,27], through α-glucosidase [28,29,30,31] and α-amylase inhibitory activities [32,33]. The consumption of Omega-3 fatty acids was able to reduce COVID-19 complications [34]. Meanwhile, sterols are known for their α-amylase and α-glucosidase inhibitory [35,36,37] and anti-viral effects [38]. More specifically, stigmasterol and β-sitosterol were able to improve glycemic regulation and stimulate pancreatic β-cells insulin secretion [39]. Several studies have also observed the activities of macroalgae lipid compounds. The fatty acids of Antarctic macroalgae have potential cytotoxic activity [40], and sterols from macroalgae have been shown to have α-glucosidase and α-amylase inhibitor activities [41]. Meanwhile, the brown macroalga *Turbinaria ornata* contains sterols that have cytotoxicity effects on cancer cells [42,43,44]. Several other macroalgae, including *Himanthalia elongate*, *Undaria pinnatifida*, *Phorphyra* spp., *Chondus crispus*, *Cystoseira* spp., and *Ulva* spp., show anti-cholesterol activity by the reduction in the total and LDL-C levels [45] through their sterol compounds. Meanwhile, fucosterol isolated from the brown macroalga *Pelvetia siliquosa* was able to reduce hyperglycemia by 25–33% [46]. Other brown macroalgae, *Eisenia bicyclis* and *Ecklonia stolonifera*, also contain fucosterols that are able to inhibit α-glucosidase [47]. Meanwhile, other studies have shown that fucosterol from macroalgae has the ability to inhibit ACE in endothelial cells [48], which shows the potential anti-viral activity of this compound. Interestingly, COVID-19 patients with DM had a reduced risk of death after treatment with α-glucosidase inhibitors compared with other anti-DM approaches [49]. Thus, further research is needed to explore the potential usage of lipophilic compounds from marine resources as a treatment for DM and COVID-19. 

In this study, the anti-hyperglycemic efficacy of macroalgal lipophilic extracts using an alloxan-induced diabetic rat model and their anti-oxidant activity were assessed. A Gas Chromatography–Mass Spectrometry (GC-MS)-based untargeted metabolomics analysis on the lipophilic extracts of *S. cristaefolium*, *T. cylindrica*, and *U. lactuca* was also performed. This was followed by evaluating the activity of the identified compounds against anti-DM and anti-COVID-19 protein targets using molecular docking and bioinformatics analysis.

## 2. Materials and Methods

### 2.1. Sample Collection, Preparation, and Lipophilic Compounds Extraction

The aim of this study was to compare the differences in the activity of three classes of macroalgae found in an abundant amount on the Lombok, Indonesia, coast, namely, Phaeophyta (brown macroalgae), represented by *S. cristaefolium*; Rhodophyta (red macroalgae), represented by *T. cylindrica*; and Chlorophyta (green macroalgae), represented by *U. lactuca*. The macroalgae were sourced from the North Lombok, West Nusa Tenggara, Indonesia, coast. The collection of the macroalgae samples was performed when the tide was low and only healthy macroalgae were used in this study. The collected macroalgae samples were identified by referring to electronic algae databases [50] and other literature (*S. cristaefolium* based on the description in [51], *T. cylindrica* based on the description in [52], and *U. lactuca* based on the description in [53]). 

The collected macroalgae samples were immediately cleaned using clean seawater. These samples were then air-dried for three days followed by dehydration at 30 °C for 24 h. Once dry, the samples were ground to achieve an optimal particle size of 40 µm. This particle size was recommended for macroalgae [54]. The powdered samples underwent lipid extraction using chloroform:methanol:water (2:1:0.5). Initially, the powdered samples were soaked with the solvent mixture and mixed for 5 min at 4 °C. This was then followed by sonication for 15 min. After 15 min, the mixture was centrifugated at 5000 rpm, 2 °C for 30 min, leading to the formation of two phases. The organic phase, which contains lipophilic compounds was then collected and then filtered. This extraction was performed three times, as this was the most sufficient number of replicates required for the extraction method, and the extracts from each run were combined. The solvent was evaporated for approximately 3 h at below 30 °C on a water bath where the sample was placed on ceramic porcelain covered with pierced aluminum foil to avoid oxidation and light denaturation of the compounds. The obtained thick extract was then allocated for further analysis.

### 2.2. In Vivo Anti-Diabetes Analysis in an Alloxan-Induced Diabetic Mouse Model

Male Wistar rats (*Rattus norvegicus*) with an average weight ranging from 200 to 250 g were used as the model animals in this study; all the rats were housed in ventilated cages and had free access to rat food and clean water. The rats were obtained from the Animal Laboratory Facility, Faculty of Medicine, University of Mataram, Indonesia. This research received ethical permission from the Medical Research Ethics Committee, Faculty of Medicine, University of Mataram, Indonesia (Approval No:285/UN18.F7/ETIK/2022), and all the experiments on the animals were performed based on the guidelines of the proper care and use of laboratory animals. The rats were classified into six groups (*n* = 4 per group): (1) untreated rats; (2) diabetic rats treated with the vehicle (0.5% CMC-Na with NaCl solution); (3) diabetic rats treated with glibenclamide (diluted in 0.5% CMC-Na with NaCl solution); (4) diabetic rats treated with *S. cristaefolium* lipophilic extract; (5) diabetic rats treated with *T. cylindrica* lipophilic extract; and (6) diabetic rats treated with *U. lactuca* lipophilic extract. All the tested samples were diluted in 0.5% CMC-Na with NaCl solution. Before being treated, the rats underwent an acclimatization phase for one week, where they were given standard rat food and clean water. Next, hyperglycemia was induced in Groups 2 to 6 by administering two intraperitoneal injections of alloxan monohydrate (150 mg/kgBW) (Sigma Aldrich, Gillingham, UK) for two consecutive days. The fasting blood glucose levels were assessed before induction and 72 days post alloxan induction using the Easy-Touch-GCU device (Bioptik Technology, Inc., Jhonghua, Taiwan). Only the rats showing fasting blood glucose levels exceeding 126 mg/dL were categorized as diabetic [55,56] and were included in the next phase of the study.

Post confirmation of the hyperglycemia status, the treatment was performed as follows: the rats in Group 1 received only food and water; Group 2 was given a vehicle solution; Group 3 was given glibenclamide orally at a dose of 0.45 mg/kgBW; and the rats in Groups 4 to 6 were treated orally with lipophilic extracts at a daily dose of 100 mg/kgBW, respectively. The fasting blood glucose was measured on days 0, 5, 10, and 15 after treatment. The recorded data underwent a statistical analysis using a homogeneity test followed by a two-way ANOVA with Dunnett’s multiple comparison test to confirm the significant differences in the fasting blood glucose levels between the treated and control diabetic rats. GraphPad Prism version 9 was used for the statistical analysis.

### 2.3. In Vitro Anti-Oxidant Analysis

The anti-oxidant activity of the macroalgae samples was evaluated using the 2,2-diphenyl-1-picryl-hydrazyl-hydrate (DPPH) and 2,2′-azino-bis(3-ethylbenzothiazoline-6-sulfonate) (ABTS) assays. One-way ANOVA was carried out on GraphPad Prism version 9 to determine the significant differences in the anti-oxidant IC_50_ values between the samples.

#### 2.3.1. DPPH Assay

The DPPH assay was performed following the method described in [57], with some modifications. The lipophilic extract was subjected to serial dilution. For every 100 μL of the sample and blank (ethanol), 100 μL of the 200 μM DPPH solution (Sigma Aldrich, Gillingham, UK) was added. This procedure was carried out in triplicate. The anti-oxidant activity was determined by

(1) $$Scavenging\;effect\;(\%)=1-(\frac{\left(Acontrol-Asample\right)}{Acontrol})\times 100$$

where the *Scavenging effect* is a percentage that shows how effective the sample is in neutralizing free radicals. *Acontrol* represents the control absorbance, which usually contains all reagents except the sample being tested. It provides basic absorption without any anti-oxidant activity. *Asample* is the absorbance of the tested sample when mixed with a DPPH solution. By subtracting the sample absorbance from the control absorbance, we obtain a measure of how much the sample has reduced the initial amount of DPPH radicals, indicating its anti-oxidant ability.

#### 2.3.2. ABTS Assay

The scavenging activity of the lipophilic extracts against the ABTS radical cations was assessed following the method described in [58], with minor modifications. Stock solutions were prepared using 7 mM ABTS in water and 2.4 mM potassium persulfate. To prepare the working solution, both stock solutions in equal volumes were mixed and incubated for 16 h at room temperature in the dark. This solution was then diluted by combining 250 μL of the ABTS mixture with 12 mL of ethanol, aiming to achieve an absorbance of approximately 0.700 units at 734 nm as measured with a spectrophotometer. For the test, 1 mL of samples at various concentrations was combined with 1 mL of dilute ABTS solution. After incubation for 7 min at room temperature, the absorbance was measured at 734 nm. The ABTS scavenging capacity of the extract was determined using Equation (1).

### 2.4. Untargeted Gas Chromatography-Mass Spectrometry (GC-MS) Metabolomics Analysis

The lipophilic extracts from *S. cristaefolium*, *T. cylindrica*, and *U. lactuca* that were obtained by extraction (Section 2.1) were then analyzed using GC-MS. For the sample preparation, the extract was diluted in chloroform:methanol, mixed in a microtube, vortexed, and centrifuged at 9500 rpm for 3 min. The resulting supernatant was transferred to a GC vial and injected into a Shimadzu GCMS-QP2010S instrument (Shimadzu Corporation, Kyoto, Japan). A 30 m long Agilent HP-5MS UI column was used. The carrier gas was Ultra High Purity Helium (He), with the settings including an injector temperature of 250 °C, a split flow of 510 mL/min, a front inlet flow of 1.00 mL/min, an MS transfer line temperature of 300 °C, and an ion source temperature of 280 °C. The compound identities were analyzed by comparing the observed properties with the properties in the NIST MainLib Library.

### 2.5. In Silico Analysis of Anti-Diabetic Activity and Anti-COVID-19 Activity

**Figure 1:**
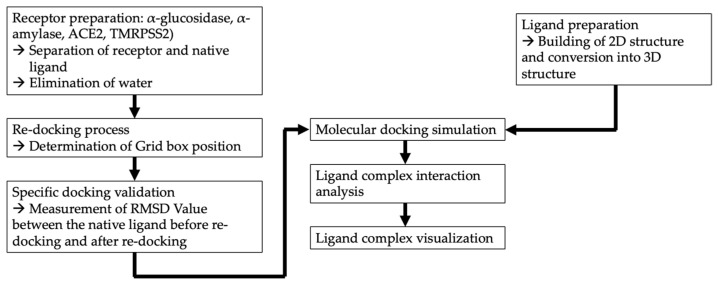
Workflow of in silico molecular docking analysis.

Molecular docking studies were carried out in silico to test the impact of the lipophilic compounds identified from the macroalgae extracts on the protein target receptors associated with DM and COVID-19. These receptors include α-amylase, α-glucosidase, angiotensin-converting enzyme 2 (ACE2), and transmembrane serine protease 2 (TMRPSS2). The respective native ligand for α-amylase and α-glucosidase was acarbose, for ACE2 it was captopril, and for TMRPSS2 it was nafamostat. The receptor preparation was carried out using DS BIOVIA Discovery Studio 2016 [59]. The ligand preparation includes creating a 2D structure using ChemDraw Ultra [42] and converting it to a 3D structure using Chem3D Pro [60]. During the re-docking phase, the grid box size was set using AutoDockTools [61], and the molecular docking simulations were performed with AutoDock Vina [62]. An analysis of the receptor–ligand complex interactions was performed with DS BIOVIA Discovery Studio 2016 [59], and the visualization of the binding sites and interactions between the ligand and receptor was facilitated by PyMOL [63]. The drug similarity scores and oral bioavailability, based on Lipinski’s rule, were determined using SWISS ADME tool (http://www.swissadme.ch (accessed on 1 April 2023). Figure 1 outlines this workflow.

## 3. Results

### 3.1. In Vivo Anti-Hyperglycemic Activity of Sargassum Cristaefolium, Tricleocarpa cylindrica, and Ulva lactuca Lipophilic Extract

A preliminary in vivo assay was performed to evaluate the anti-hyperglycemic potential of the macroalgae lipophilic extracts, which is an important aspect in the management of DM. The differences in the activity of the three classes of macroalgae, namely, Phaeophyta, Rhodophyta, and Chlorophyta, were observed. We aimed to choose the species that were found abundantly on the Lombok, Indonesia, coast. *S. cristaefolium* was chosen to represent Phaeophyta, *T. cylindrica* to represent Rhodophyta, and *U. lactuca* to represent Chlorophyta.

**Figure 2:**
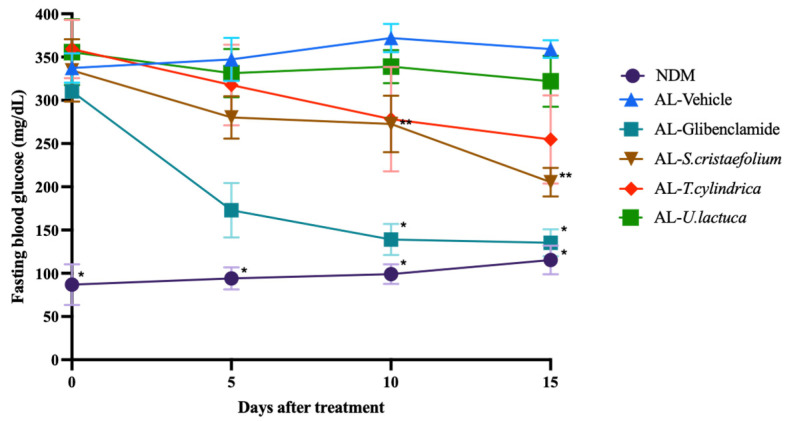
In vivo anti-hyperglycemic analysis of *Sargassum cristaefolium*, *Tricleocarpa cylindrica*, and *Ulva lactuca* on alloxan-induced diabetic rats. NDM = non-diabetes mellitus group; AL = alloxan-induced diabetic rats. Two-way ANOVA analysis with Dunnett’s multiple comparison test, * *p*-value < 0.05; ** *p*-value < 0.01; significance toward Alloxan-induced diabetic rats.

The rats, after receiving two intraperitoneal doses of alloxan monohydrate (150 mg/kgBW) for two days, showed an increase in the fasting blood sugar levels ranging from approximately 300 to 350 mg/dL (Figure 2). This increase was significantly greater than that observed in the untreated rats. These hyperglycemic rats were then chosen as a DM model and given glibenclamide (0.45 mg/kgBW) and lipophilic macroalgae extract (100 mg/kgBW) every day orally for 15 days.

A decrease in the fasting blood glucose was clearly visible in the rats treated with glibenclamide on day 5 post-treatment and continued to decrease until day 15. In contrast, treatment with the macroalga *S. cristaefolium* lipophilic extract caused a significant reduction in the fasting blood sugar levels on day 10, and this trend was maintained until day 15 after treatment. Meanwhile, another macroalga, *T. cylindrica,* did reduce the blood glucose levels on day 15 in the alloxan-induced rat model; however, the effect was not as significant as *S. cristaefolium*. Meanwhile, *U. lactuca* showed lower anti-hyperglycemia activity compared to the other macroalgae, *S. cristaefolium* and *T. cylindrica* (Figure 2). These preliminary findings demonstrate the anti-hyperglycemic properties of the lipophilic extracts.

### 3.2. In Vitro Anti-Oxidant Activity of Sargassum cristaefolium, Tricleocarpa cylindrica, and Ulva lactuca Lipophilic Extract

The in vitro anti-oxidant studies revealed that the lipophilic extracts of *S. cristaefolium*, *T. cylindrica*, and *U. lactuca* exhibited anti-oxidant activity even though the activity was considered a low-potential anti-oxidant. Specifically, they showed IC_50_ values of 206.7 ± 0.11 µg/mL, 252 ± 0.10 µg/mL, and 308.6 ± 0.13 µg/mL, respectively, as determined by the DPPH assay. Meanwhile, the ABTS assay produced IC_50_ values of 200.47 ± 0.089 µg/mL, 353.22 ± 0.070 µg/mL, and 386.42 ± 0.050 µg/mL for the same extract. Ascorbic acid was used as a positive control for the assay and showed IC_50_ values of 3.667 ± 0.02 µg/mL (DPPH assay) and 5.58 ± 0.02 µg/mL (ABTS assay) as summarized in Table 1, showing that the method performed well. The IC_50_ values of the macroalgae lipophilic extracts obtained in this study are lower than ascorbic acid but are still considered acceptable and in the normal range for crude macroalgae extracts, as shown in other studies. The acetone extracts of the macroalga *Cystoseira amantacea* showed an IC_50_ value of 408.81 μg/mL with the DPPH method [64]. Meanwhile, another study has also shown that the ethyl acetate extract of *Sargassum angustifolium* had an anti-oxidant IC_50_ value of 400 μg/mL by using the ABTS method [65]. This is due to the fact that the macroalgae lipophilic extract is still a whole crude extract and not a single isolated compound.

The lipophilic extract of *S. cristaefolium* had superior anti-oxidant activity compared to *T. cylindrica* and *U. lactuca*. This is in line with the anti-hyperglycemic data where the lipophilic extract of *S. cristaefolium* produced a more pronounced reduction in fasting blood glucose, followed by *T. cylindrica*, and then *U. lactuca* showed the least effective. These observations suggest that the anti-hyperglycemic action of *S. cristaefolium* lipophilic extract may be mediated through the reduction in ROS. Contemporary advances in metabolic research have identified that molecules with anti-oxidant capacity can influence blood glucose concentrations and monitor changes in biochemical indicators related to glucose metabolism [66]. Anti-oxidants may improve insulin sensitivity and modulate carbohydrate metabolism [67]. In addition, pioglitazone, a known type-2 anti-diabetic agent and peroxisome proliferator-activated receptor agonist, regulates glucose regulation through its anti-oxidant activity. Thus, entities with anti-oxidant attributes show promise in their potential as anti-diabetic agents. In addition, the solid anti-oxidant activity of *S. cristaefolium* may be due to the synergy of several bioactive compounds, not just the identified lipophilic molecules. It is important to understand that although these findings are significant, further isolation and testing of these compounds is necessary to decipher the exact mechanisms underlying their anti-oxidant properties.

### 3.3. Untargeted GC-MS Metabolomics Analysis of Macroalgae Lipophilic Extract

**Figure 3:**
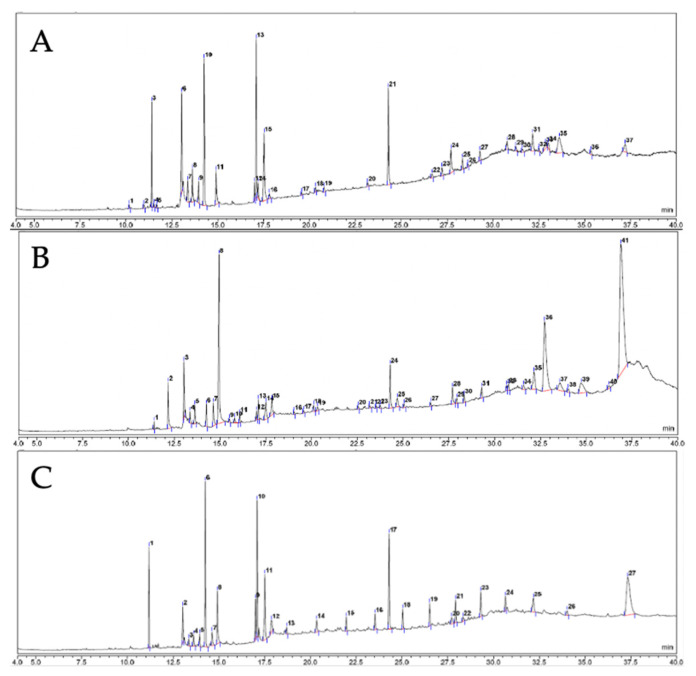
Gas Chromatography–Mass Spectrometry (GC-MS) chromatogram of (**A**) *Sargassum cristaefolium* lipophilic extract; (**B**) *Tricleocarpa cylindrica* lipophilic extract; and (**C**) *Ulva lactuca* lipophilic extract.

Performing a phytochemical analysis of the compounds in extracts helps identify new compounds with potential bioactivity. Therefore, in this study, after verifying the anti-hyperglycemic and anti-oxidant properties of the lipophilic macroalgae extracts, the metabolites in the extracts were analyzed. A GC-MS-based untargeted metabolomics approach was used to profile the compounds present in the lipophilic extracts of the three macroalgae: a total of 37 peaks were observed in *S. cristaefolium*, 41 peaks were observed in the *T. cylindrica* extract, and 37 peaks were observed in the *U. lactuca* extract. Figure 3 displays the GC-MS spectrum. Each peak was identified using the NIST MainLib database by referring to the retention time, molecular formula, and molecular weight.

Based on NIST MainLib identification, 16 lipophilic compounds were detected in the *S. cristaefolium* extract, 20 in the *T. cylindrica* extract, and 14 in the *U. lactuca* extract. Most of the lipophilic compounds detected in the macroalgae belong to the family of fatty acids, terpenes, and steroids. For *S. cristaefolium*, the dominant compound is ethyl iso-allocholate, a steroid. In addition, several fatty acids were identified. In *T. cylindrica*, the main compounds are steroids. Other major compounds in the *T. cylindrica* lipophilic extracts were fatty acids. Meanwhile, for *U. lactuca*, the main compound is cholest-5-en-3-ol,24-propylidene-, (3β)-, a steroid. Other fatty acids were also detected. Detailed data on the compounds detected in the lipophilic extracts of these macroalgae are presented in Table 2.

### 3.4. In Silico Molecular Docking Analysis

Natural products including marine metabolites offer potential treatments for a variety of human ailments. However, challenges in analyzing large numbers of compounds in the laboratory have hampered progress in the development of medicinal compounds based on these natural products. As a result, computational analysis has become an invaluable tool in drug development, especially when working with natural resources rich in metabolites. Virtual screening methods are often used to reduce the costs and time associated with developing natural product-based drugs. Specifically, more negative values of the total net charge indicate a higher binding affinity [68].

An in silico molecular docking technique was used in this study to screen the lipophilic compounds of macroalgae (*S. cristaefolium*, *T. cylindrica*, and *U. lactuca*), which were identified via an untargeted GC-MS metabolomics analysis. These compounds were evaluated to determine their potential ligand binding affinity toward established anti-DM and COVID-19 targets, specifically α-glucosidase, α-amylase, ACE2, and TMPRSS2. Compounds that have the ability to inhibit α-glucosidase and α-amylase activity could cause an inhibitor of sugar or carbohydrate absorption and digestion from the intestine, which then causes the reduction in post-prandial blood glucose spikes. Consequently, inhibitors of this enzyme may have therapeutic potential in the management of DM [69]. In the context of COVID-19, compounds that inhibit ACE2 have the potential to prevent the virus infection. This is because the binding of the virus SARS-CoV-2 S-protein to the ACE2 receptor initiates viral entry, which then could cause viral replication and viral spread [70]. In addition to inhibiting ACE2, other characteristics of a potential anti-COVID-19 agent is the ability to inhibit ACE2′s co-receptor known as TMPRSS2, which has the role of facilitating viral entry into host cells [71,72,73,74].

**Figure 4:**
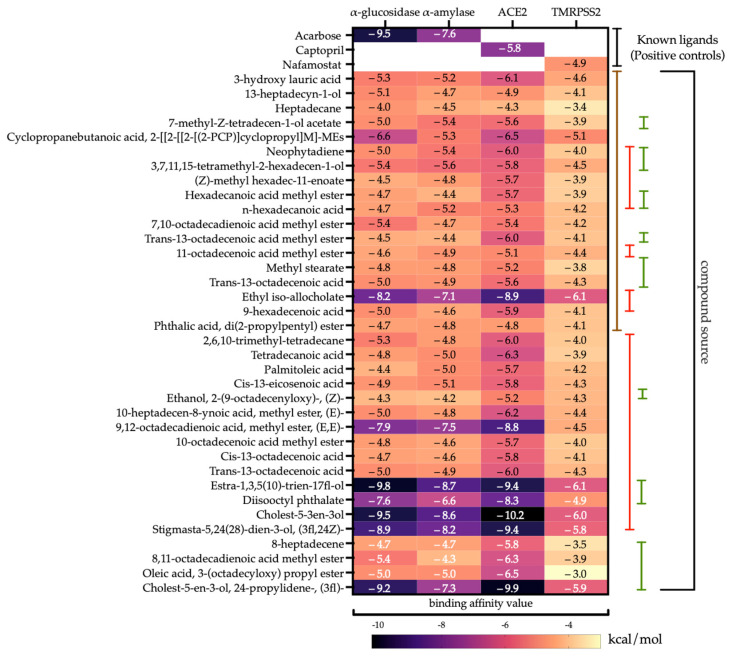
Binding affinity value of *Sargassum cristaefolium*, *Tricleocarpa cylindrica*, and *Ulva lactuca* lipophilic compounds toward anti-DM and anti-COVID-19 target proteins (the colored lines represent the macroalgae—brown line: *Sargassum cristaefolium*; red line: *Tricleocarpa cylindrica*; and green line: *Ulva lactuca*).

Our findings indicate that several lipophilic compounds from *S. cristaefolium*, *T. cylindrica*, and *U. lactuca* have the ability to bind and inhibit proteins associated with either DM or COVID-19 and even both (Figure 4). Notably, its binding affinity exceeds that of the reference compound (native ligand or positive control). For example, ethyl iso-allocholate, present in the lipophilic extracts of *S. cristaefolium* and *T. cylindrica*, showed effective binding and inhibition of ACE2 and TMPRSS2. It also showed effective binding toward α-glucosidase and α-amylase, but the activity was slightly lower than the positive controls. This indicates that the anti-hyperglycemic activity of the *S. cristaefolium*, as shown in the in vivo hyperglycemic study, could be due to other mechanisms, possibly through the reduction in ROS, as the *S. cristaefolium* had the highest anti-oxidant activity compared to the other macroalgae (Table 1).

It should be noted that many factors, including the presence of other bioactive compounds or possible antagonistic effects, can influence differences in the activity between macroalgae extracts. The fact that *U. lactuca* only shows ACE2 inhibitory activity and no potential binding affinity toward anti-DM targets suggests that its chemical profile may differ significantly from other macroalgae or have specific compounds that target specific pathways. The in silico molecular docking results were also in line with the anti-hyperglycemic activity analysis, which shows that *U. lactuca* did not show potential anti-hyperglycemic properties. In contrast, steroid derivatives that were only found in *T. cylindrica*, e.g., estra-1,3,5(10)-trien-17-β-ol-17-α-butadinyl-3-methoxy; 17-(1,5-dimethylhexyl)-10,13-dimethyl-2,3,4,7,8,9,10,11,12,13,14,15,16,17-tetradecahydro-1H-cyclopenta[a]phenanthren-3-ol, and stigmasta-5,24(28)-dien-3-ol, (3β,24Z)-, showed the ability to bind to both anti-DM and anti-COVID-19 target proteins, i.e., α-glucosidase, α-amylase, ACE2, and TMPRSS2. Interestingly, its binding affinity exceeds the reference standard (Figure 4).

**Figure 5:**
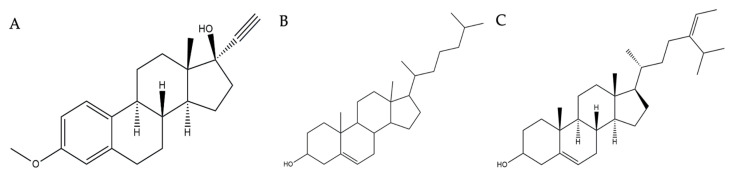
Chemical structure of compounds detected in red macroalga *Tricleocarpa cylindrica* with anti-diabetes mellitus and anti-COVID-19 activities: (**A**) Compound **1:** Estra-1,3,5(10)-trien-17-*β*-ol-17-α-butadinyl-3-methoxy, (**B**) Compound **2:** 17-(1,5-dimethyl)-10,13-dimethyl-2,3,4,7,8,9,10,11,12,13,14,15,16,17,tetradecahydro-1H-cyclopenta(a)phenanthrene-3-ol, and (**C**) Compound **3:** Stigmasta-5,24(28)-dien-3-ol, (3*β*,24Z)-.

**Figure 6:**
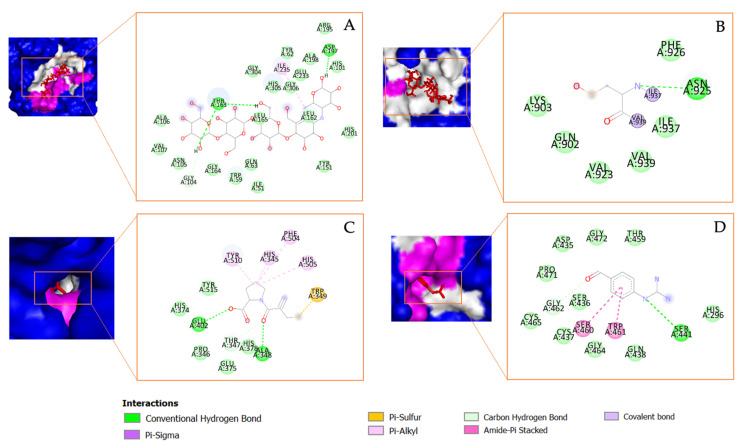
The molecular docking results and the chemical bond ligand–protein interaction of native ligands (positive controls) with the target proteins: (**A**) acarbosa with α-amylase; (**B**) acarbosa with α-glucosidase; (**C**) captopril with ACE2; and (**D**) nafamostat with TMRPSS2.

The chemical structure of the potential compounds detected in *Tricleocarpa cylindrica* can be observed in Figure 5. Estra-1,3,5(10)-trien-17-β-ol-17-α-butadinyl-3-methoxy has been reported to possess several biological activities, including anti-arrhythmic activities [75], anti-fungal activity [76], and activity toward SARS-CoV-2-related protein targets [77]. The next compound, 17-(1,5-dimethylhexyl)-10,13-dimethyl-2,3,4,7,8,9,10,11,12,13,14,15,16,17-tetradecahydro-1H-cyclopenta[a]phenanthren-3-ol, is also found to have activity in treating mineral disorders [78], fungal infections [79], bacterial infections, and an increase in ROS production [80]. Meanwhile, the third compound, stigmasta-5,24(28)-dien-3-ol, (3β,24Z)-, has been shown to have anti-oxidant activity [81], anti-diabetes activity [82], anti-tumor activity [83], anti-cervical cancer activity [84], and anti-bacterial activity [85]. Figure 6 visualizes the interaction between the native ligand (serving as a positive control) and the corresponding protein target. All native ligands form strong hydrogen bonds with the amino acid residues of their specific protein targets: acarbose with α-amylase and α-glucosidase, captopril with ACE2, and nafamostat with TMPRSS2. In addition to these interactions, various other types of bonds, such as Pi–Alkyl, Pi–Sulfur, and Amide–Pi Stacking, were also observed. This indicates that these native ligands have a strong binding affinity with the protein targets.

**Figure 7:**
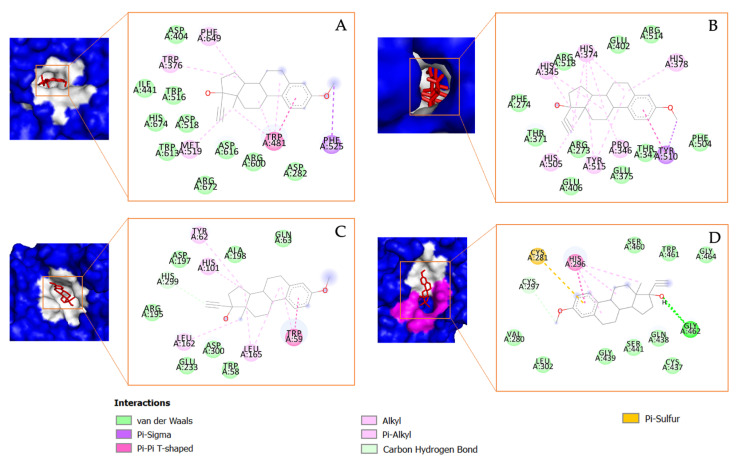
The molecular docking results and the chemical bond ligand–protein interaction of Estra-1,3,5(10)-trien-17-β-ol-17-α-butadinyl-3-methoxy (Compound **1**) detected in *Tricleocarpa cylindrica* against the diabetes mellitus (DM) and COVID-19-related proteins (**A**) α-glucosidase, (**B**) α-amylase, (**C**) ACE2, and (**D**) TMRPSS2.

Estra-1,3,5(10)-trien-17-β-ol-17-α-butadinyl-3-methoxy, which will be referred to as Compound **1** in this study, also formed several interactions with the receptors, including with the amino acid residues of α-glucosidase, including Pi–Alkyl interactions with Phe^649^, Trp^376^, and Met^519^; the Pi–Sigma interaction with Phe^525^; and the Pi–Pi T-shaped interaction with Trp^481^. Several Pi–Alkyl bonds were also observed between the compound and α-amylase amino acid residues, including His^345^, His^374^, His^378^, His^505^, Tyr^515^, and Pro^346^. A Pi–Sigma was also observed between Compound **1** and Tyr^510^. Meanwhile, for COVID-19-related targets, such as ACE2, Compound **1** formed a Pi–Alkyl bond with Tyr^62^, His^101^, Leu^162^, and Leu^165^ and a Pi–Pi T-shaped bond with Trp^59^. The compound also formed several bonds with TMRPSS2, including a hydrogen bond with Gly^462^, a Pi–Pi T-shaped bond with His^296^, and a Pi–sulfur bond with Cys^281^, as shown in Figure 7.

**Figure 8:**
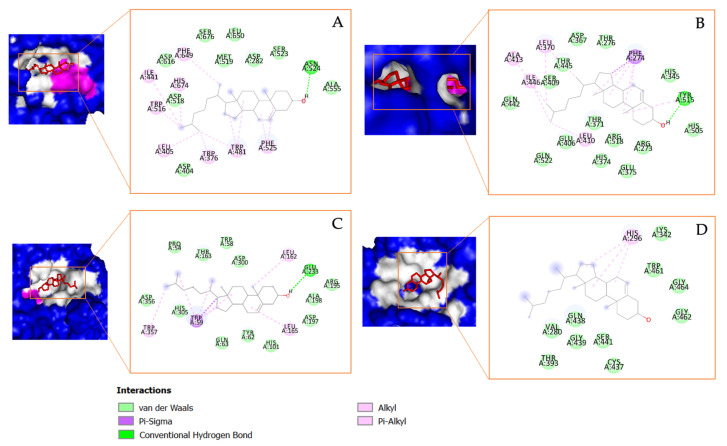
The molecular docking results and the chemical bond ligand–protein interaction of 17-(1,5-dimethylhexyl)-10,13-dimethyl-2,3,4,7,8,9,10,11,12,13,14,15,16,17-tetradecahydro-1H cyclopenta[a]phenanthren-3-ol (Compound **2**) detected in *Tricleocarpa cylindrica* against the diabetes mellitus (DM) and COVID-19-related proteins (**A**) α-glucosidase, (**B**) α-amylase, (**C**) ACE2, and (**D**) TMRPSS2.

Another important steroid derivative compound detected in *T.cylindrica* was 17-(1,5-dimethylhexyl)-10,13-dimethyl-2,3,4,7,8,9,10,11,12,13,14,15,16,17-tetradecahydro-1H cyclopenta[a]phenanthren-3-ol, which will be referred to as Compound **2** in this study, and it shows a high binding affinity to α-glucosidase, α-amylase, ACE2, and TMPRSS2. For α-glucosidase, this compound interacts via the hydrogen bonds with Asn^524^ and forms several Pi–Alkyl bonds with the amino acid residues, including Phe^649^, His^674^, Ile^441^, Trp^516^, Leu^405^, Trp^376^, Trp^481^, and Phe^525^. In its interaction with α-amylase, a hydrogen bond is formed with Tyr^515^, complemented by a Pi–Sigma bond with Phe^274^ and several Pi–Alkyl bonds. With ACE2, the compound forms a strong hydrogen bond with amino acid Glu^233^ and has a Pi–Sigma bond with Trp^59^. Pi–Alkyl bonds were also observed with residues Leu^162^, Leu^165^, and Trp^359^. Regarding TMPRSS2, a Pi–Alkyl bond with His^296^ was observed, as visualized in Figure 8.

**Figure 9:**
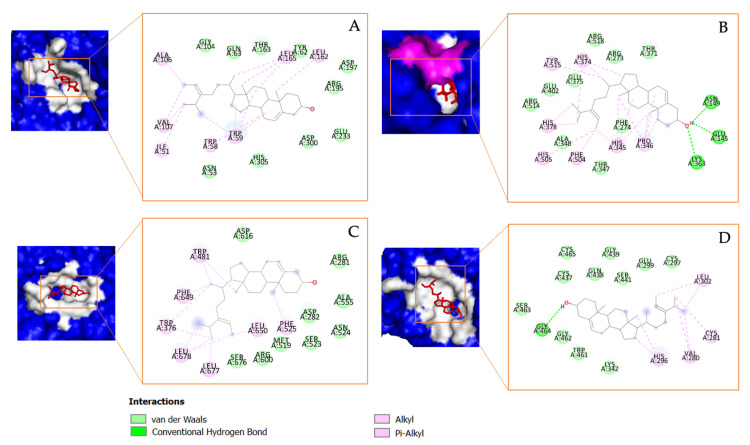
The molecular docking results and the chemical bond ligand–protein interaction of Stigmasta-5,24(28)-dien-3-ol,(3*β*,24Z)- (Compound **3**) in *Tricleocarpa cylindrica* against the diabetes mellitus (DM) and COVID-19-related proteins (**A**) α-glucosidase, (**B**) α-amylase, (**C**) ACE2, and (**D**) TMRPSS2.

A third compound identified in *T. cylindrica*, i.e., Stigmasta-5,24(28)-dien-3-ol,(3*β*,24Z)-, which will be referred to as Compound **3** in this study, showed a clear binding affinity to all the protein targets. For α-glucosidase, this compound forms several Pi–Alkyl bonds with the amino acid residues, including Leu^162^, Leu^165^, Ala^106^, Val^107^, Trp^58^, and Trp^59^. In its interaction with α-amylase, it forms hydrogen bonds with residues Asn^149^, Glu^145^, and Lys^363^. Additionally, Pi–Alkyl bonds were observed with Tyr^515^, His^345^, His^374^, His^378^, His^505^, Phe^504^, and Pro^346^. In further interactions, this compound forms Pi–Alkyl bonds with Trp^376^, Trp^481^, Phe^649^, Leu^650^, Leu^677^, and Leu^678^. With TMPRSS2, a hydrogen bond is formed with Gly^464^. Pi–Alkyl bonds are also seen with residues Leu^302^, Cys^281^, Val^280^, and His^296^. This interaction is visualized in Figure 9.

### 3.5. Analysis of Physiochemical Properties and ADME Studies of the Compounds

Three lipophilic compounds were identified due to their strong binding affinity to DM and COVID-19-related protein targets, Compound **1**, **-2**, and **-3**. To measure their potential as a drug, its physicochemical properties were evaluated based on Lipinski’s rule of five. The viability of a compound as a drug increases if it meets these criteria and no more than one physicochemical criterium is violated [86]. Compound **1** fully complies with Lipinski’s rule, as it does not violate any criteria. All properties, including the molecular weight, hydrogen bond donor, hydrogen bond acceptor, log P value, and molar refractivity, were within the accepted range. Compound **2**, although displaying a log P value exceeding 5, indicating high lipophilicity, can still be considered for further drug discovery and development because it has only one violation of Lipinski’s rule of five. Meanwhile, based on this analysis, Compound **3** violates two criteria: the log P value was higher than 5 and the molar refractivity exceeded 130. Therefore, Compound **3** would need to be developed into a certain drug delivery system in order to have higher oral bioavailability and to be used as a drug. Detailed data regarding the physicochemical properties of these compounds are tabulated in Table 3.

**Figure 10:**
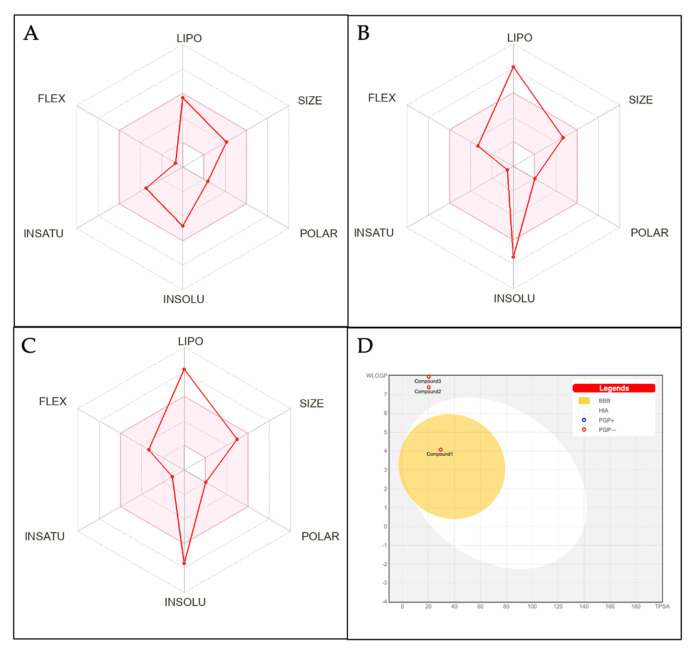
Bioavailability radar and boiled-egg diagram of the compounds’ physicochemical properties. (**A**) Compound **1**: Estra-1,3,5(10)-trien-17-*β*-ol-17-α-butadinyl-3-methoxy; (**B**) Compound **2**: 17-(1,5-Dimethylhexyl)-10,13-dimethyl-2,3,4,7,8,10,11,12,13,14,15,16,17-tetradecahydro-1H-cyclopenta[a]phenanthren-3-ol; (**C**) Compound **3**: Stigmasta-5,24(28)-dien-3-ol, (3*β*,24Z)-; and (**D**) boiled-egg diagram of all three compounds.

The oral bioavailability of a compound is an important criterion for drug likeness and can be predicted using a bioavailability radar [87]. The radar, depicted in Figure 10A–C, evaluates the suitability of compounds for oral consumption. The optimal range for each property, such as the lipophilicity, size, polarity, solubility, saturation, and flexibility, is represented by the pink area. The results of the following compounds show that the Compound **1** properties were all within the optimal pink area. Meanwhile, Compound **2** and Compound **3** were partly in the pink area. All these compounds qualify as drug-like. However, Compound **2** and **-3** require extra consideration during drug formulation due to their high lipophilicity, which was also evidenced by their log P value, which exceeded 5, summarized in Table 3. Many currently emerging drug candidates exhibit high lipophilicity. For example, novel tuberculosis drugs that were lipophilic performed better than less lipophilic drugs [88,89]. Therefore, despite their lipophilicity, Compound **2** and **-3** showed potent activity against target proteins, thus underlining their potential as drug candidates.

The pharmacokinetic profile of candidate compounds can be assessed using a boiled-egg diagram, as shown in Figure 10D. This diagram evaluates passive gastrointestinal absorption (HIA) and brain penetration (BBB). The white part indicates a greater possibility of passive absorption in the gastrointestinal tract, while the yellow part indicates a greater possibility of penetration into the brain. Red dots indicate that the compounds are not affected by P-glycoprotein-mediated extrusion from the CNS. These findings revealed that all compounds were not substrates for P-gp. Compound **1** is located in the yellow area, indicating the possibility of being passively absorbed by the gastrointestinal tract and it can penetrate the BBB. In contrast, Compound **2** and **-3** were located in the gray area, indicating that they may not be efficiently absorbed by the gastrointestinal tract or penetrate the BBB. These results may originate from its structural complexity or increased lipophilicity. Addressing these challenges may require customized delivery systems to target specific regions.

The intricate relationship between the structure of the lipophilic compounds and their binding affinity to DM and COVID-19 protein targets offers promising avenues in drug design. Moreover, variations in Lipinski’s rule of five violations among compounds underscore the complexity of translating molecular properties into drug-like behavior. These findings emphasize the need for a multidimensional approach when evaluating potential drug candidates, including considering traditional criteria such as Lipinski’s rule of five and innovative methods, such as the bioavailability radar.

## 4. Discussion

The main aim of this study was to explore the potency of macroalgae lipophilic compounds as drug candidates for COVID-19 patients with DM comorbidity treatment, by using several approaches. Our findings demonstrate the importance of a multifaceted approach to drug discovery. Exploration of these compounds has significance considering the ongoing challenges faced by the global healthcare community in addressing this health problem, including limitations in performing laboratory experiments, such as analyzing a wide range of plant metabolites for COVID-19 studies where the SARS-CoV-2 virus is highly infectious to humans. The methods we used allow a rigorous and detailed assessment of the potential of these compounds as drug candidates. In this study, we integrated in vitro, in vivo, and in silico approaches to screen the large number of lipophilic metabolites in macroalgae in order to find the potential drug candidates. Moreover, using Lipinski’s rule of five criteria provides a comprehensive framework for understanding the pharmacokinetics and pharmacodynamics of these compounds. The application of these methods provides advantages that cannot be easily obtained from traditional techniques.

The analysis of the lipophilic extracts of macroalgae *S. cristaefolium*, *T. cylindrica,* and *U. lactuca* has shown that these extracts have anti-oxidant and anti-hyperglycemic properties. Recent opinions have emerged regarding the efficiency of anti-oxidant consumption and treatment of diseases in the human body, as reviewed in [90]. It is mentioned that anti-oxidants might not have a direct correlation with disease progression and treatment. However, based on other studies, it is shown that anti-oxidant consumption is an important factor in disease treatment, especially diseases that are a result of high ROS production, such as DM. Anti-oxidants play a significant role in the management of DM and could reduce hyperglycemia conditions, even though not directly. For example, dietary supplementations of anti-oxidants such as N-acetylcysteine, ascorbic acid, and α-lipoic acids are able to reduce DM complications [91]. The consumption of anti-oxidants could also reduce key biochemical changes found in hyperglycemia caused by superoxide radicals, including increased flux through the polyol pathway (glucose conversion to sorbitol) and the formation of AGE products [92,93]. Some other studies have also shown that individuals with low concentrations of anti-oxidants have a higher risk of DM complications [94,95,96]. Therefore, it could be assumed that anti-oxidants still play an important role in DM treatment.

Further, in silico molecular docking assessments have shown that three compounds detected in the *T. cylindrica* lipophilic extract, identified as steroid derivatives, have the ability to inhibit DM and COVID-19 protein targets, α-amylase, α-glucosidase, ACE2, and TMRPSS2, and their binding affinity was higher than their respective positive controls. Although all three compounds are promising (Compound **1**, Compound **2**, and Compound **3**), they present varying challenges and benefits. Compound **1** fully complies with Lipinski’s rule of five; however, the binding affinity toward all the protein targets was not as significant as Compound **2** and Compound **3**. Meanwhile, Compound **2** and Compound **3** were highly lipophilic. Therefore, this needs to be addressed by further optimization and development of the right drug delivery system to increase their oral bioavailability.

This research presents a step forward in searching for effective drug candidates against DM and COVID-19. By integrating established rules, advanced visualization tools, and rigorous analysis, we were able to navigate the complexities of marine-based drug discovery and development. The journey from a potential metabolite to a market-ready drug is still long and full of challenges. Further research including the isolation of these single compounds from the macroalgae lipophilic extract, evaluation of compound activity and toxicity through in vitro and in vivo assays, and optimization of the drug formulation are all critical steps needed to bring these promising candidate metabolites to the hand of COVID-19 with DM comorbidities patients.

## 5. Conclusions

This study revealed that the *S. cristaefolium*, *T. cylindrica,* and *U. lactuca* lipophilic extracts all possess anti-oxidant activity, but only *S. cristaefolium* and *T. cylindrica* possess anti-hyperglycemic properties. Moreover, the steroid derivative compounds in the *T. cylindrica* lipophilic extracts, known as estra-1,3,5(10)-trien-17-β-ol-17-α-butadinyl-3-methoxy;17-(1,5-dimethylhexyl)-10,13-dimethyl-2,3,4,7,8,9,10,11,12,13,14,15,16,17-tetradecahydro-1H-cyclopenta(a)phenanthrene-3-ol, and stigmasta-5,24(28)-dien-3-ol,(3β,24Z)-, were able to inhibit the DM and COVID-19-related protein targets, namely α-glucosidase, α-amylase, ACE2, and TMRPSS2. Based on the physicochemical and AMDE studies, even though the second and third compounds would need further drug formulation and delivery system improvements, these compounds are still considered potential drug candidates. The results of this study would provide a basis for further in vitro and in vivo analysis, and the process of optimization of the drug formulation and development of these potential lipophilic compounds.

## Figures and Tables

**Table 1:** IC_50_ values of anti-oxidant activity of *S. cristaefolium*, *T. cylindrica*, and *U. lactuca* lipophilic extract.

Sample	IC_50_ (μg/mL) ± SEM	IC_50_ (μg/mL) ± SEM
	DPPH	ABTS
*Sargassum cristaefolium*	206.7 ± 0.11 ^a^	200.47 ± 0.09 ^a^
*Tricleocarpa cylindrica*	252 ± 0.10 ^b^	353.22 ± 0.07 ^a,b^
*Ulva lactuca*	308.6 ± 0.13 ^b^	386.42 ± 0.05 ^a,b^
Ascorbic acid	3.67 ± 0.02	5.58 ± 0.02

^a^ shows statistical differences (*p* < 0.05) between samples and standard, ^b^ shows statistical differences (*p* < 0.05) between samples and *S. cristaefolium*.

**Table 2:** Identified compounds from Gas Chromatography–Mass Spectrometry (GC-MS) analysis of macroalgae lipophilic extract.

*Sargassum cristaefolium*						
No.	Ret. Time (min)	Compound Name	Compound Classification	Chemical Formula	Mol. Weight	Peak Area (%)
1	10.17	Dodecanoic acid, 3-hydroxy-(3-hydroxy lauric acid)	Fatty acid	C_12_H_24_O_3_	216	0.28
2	10.98	13-heptadecyn-1-ol	Fatty alcohol	C_17_H_32_O	252	0.39
3	11.4	Heptadecane	Alkane hydrocarbon	C_17_H_36_	240	5.6
4	11.53	7-methyl-Z-tetradecen-1-ol acetate	Ester	C_17_H_32_O_2_	268	0.21
5	11.69	Cyclopropanebutanoic acid, 2-[[2-[[2-[(2-pentyl cyclopropyl) methyl] methyl ester	Fatty acids methyl ester	C_25_H_42_O_2_	374	0.24
6	13.03	Neophytadiene	Terpenoid	C_20_H_38_	278	8.42
7	13.36	3,7,11,15-tetramethyl-2-hexadecen-1-ol	Terpenoid	C_20_H_40_O	296	5.49
8	13.95	(Z)-methyl hexadec-11-enoate	Fatty acid methyl ester	C_17_H_32_O_2_	268	1.64
9	14.26	Hexadecanoic acid methyl ester	Fatty acid methyl ester	C_17_H_34_O_2_	270	12.27
10	14.91	n-hexadecanoic acid	Fatty acid	C_16_H_32_O_2_	256	4.02
11	17	7,10-octadecadienoic acid, methyl ester	Fatty acid methyl ester	C_19_H_34_O_2_	294	1.52
12	17.1	Trans-13-octadecenoic acid, methyl ester	Fatty acid methyl ester	C_19_H_36_O_2_	296	13.48
13	17.19	11-octadecenoic acid, methyl ester	Fatty acid methyl ester	C_19_H_36_O_2_	296	1.39
14	17.52	Methyl stearate	Saturated methyl ester	C_19_H_38_O_2_	298	8.67
15	17.82	Trans-13-octadecenoic acid	Fatty acid	C_18_H_34_O_2_	282	0.57
16	19.57	Ethyl iso-allocholate	Steroid	C_26_H_44_O_5_	436	24.24
17	20.34	9-hexadecenoic acid	Fatty acid	C_16_H_30_O_2_	254	0.5
18	24.3	Phthalic acid, di(2-propylpentyl) ester	Lipophilic chemicals	C2_4_H_38_O_4_	390	9.24
19	37.2	Spirost-8-en-11-one, 3-hydroxy-, (3β,5a,14β,20β,22β,25R)-	Steroid	C_27_H_40_O_4_	428	1.86
***Tricleocarpa cylindrica***						
**No.**	**Ret. Time** **(min)**	**Compound Name**	**Compound Classification**	**Chemical Formula**	**Mol. Weight**	**Peak Area (%)**
1	11.4	Tetradecane, 2,6,10-trimethyl-	Terpenoid	C_17_H_36_	240	0.25
2	12.16	Tetradecanoic acid	Fatty acid	C_14_H_28_O_2_	228	2.44
3	13.03	Neophytadiene	Diterpene	C_20_H_38_	278	3.06
4	13.36	3,7,11,15-tetramethyl-2-hexadecen-1-ol	Terpenoid	C_20_H_40_O	296	1.82
5	14.25	Hexadecanoic acid, methyl ester	Fatty acid	C_17_H_34_O_2_	270	1.11
6	14.63	Palmitoleic acid	Fatty acid	C_16_H_30_O_2_	254	1.64
7	14.95	n-hexadecanoic acid	Fatty acid	C_16_H_32_O_2_	256	13.44
8	15.52	cis-13-eicosenoic acid	Fatty acid	C_20_H_38_O_2_	310	0.19
9	15.8	ethanol 2-(9-octadecenyloxy)-,(Z)-	Dialkyl ether	C_20_H_40_O_2_	312	0.39
10	16.07	10-heptadecen-8-ynoic acid, methyl ester, (E)-	Fatty acid methyl ester	C_18_H_30_O_2_	278	0.46
11	17	9,12-octadecadienoic acid, methyl ester, (E,E)-	Fatty acid methyl ester	C_19_H_34_O_2_	294	0.46
12	17.09	10-octadecenoic acid, methyl ester	Fatty acid methyl ester	C_19_H_36_O_2_	296	1.04
13	17.51	11-octadecenoic acid, methyl ester	Fatty acid methyl ester	C_19_H_36_O_2_	296	1.73
14	17.83	cis-13-octadecenoic acid	Fatty acid	C_18_H_34_O_2_	282	1.39
15	19.05	9-hexadecenoic acid	Fatty acid	C_16_H_30_O_2_	254	0.5
16	19.57	trans-13-octadecenoic acid	Fatty acid	C_18_H_34_O_2_	282	0.2
17	20.11	Estra-1,3,5(10)-trien-17-β-ol-17-α-butadinyl-3-methoxy	Steroid	C_18_H_24_O	256	0.43
18	22.55	Ethyl iso-allocholate	Steroid	C_26_H_44_O_5_	436	14.67
19	23.17	cis-13-eicosenoic acid	Fatty acid	C_20_H_38_O_2_	310	0.22
20	24.29	Diisooctyl phthalate	Phtalate ester	C_24_H_38_O_4_	390	2.84
21	32.73	17-(1,5-dimethylhexyl)- 10,13-dimethyl 2,3,4,7,8,9,10,11,12, 13,14,15,16,17- tetradecahydro- 1H- cyclopenta[a] phenanthren-3-ol	Steroid	C_27_H_46_O	386	15.15
22	36.89	Stigmasta-5,24(28)-dien-3-ol, (3β,24Z)-	Steroid	C_29_H_48_O	412	36.59
***Ulva lactuca***						
**No.**	**Ret. Time** **(min)**	**Compound Name**	**Compound Classification**	**Chemical Formula**	**Mol. Weight**	**Peak Area (%)**
1	11.8	8-Heptadecene	Fatty alcohol	C_17_H_34_	238	4.93
2	13.03	Neophytadiene	Terpenoid	C_20_H_38_	278	4.93
3	13.36	Ethanol, 2-(9-octadecenyloxy)-, (Z)-	Dialkyl ether	C_20_H_40_O_2_	312	0.69
4	13.62	3,7,11,15-tetramethyl-2-hexadecen-1-ol	Terpenoid	C_20_H_40_O	296	1.15
5	13.95	(Z)-methyl hexadec-11-enoate	Fatty acid methyl ester	C_17_H_32_O_2_	268	0.89
6	14.26	Hexadecanoic acid, methyl ester	Fatty acid methyl ester	C_17_H_34_O_2_	270	12.58
7	14.63	9-hexadecenoic acid	Fatty acid	C_16_H_30_O_2_	254	1.48
8	14.91	n-hexadecanoic acid	Fatty acid	C_16_H_32_O_2_	256	5.23
9	16.99	8,11-octadecadienoic acid, methyl ester	Fatty acid methyl ester	C_19_H_34_O_2_	294	3.01
10	17.09	trans-13-octadecenoic acid, methyl ester	Fatty acid methyl ester	C_19_H_36_O_2_	296	10.78
11	17.51	Methyl stearate	Fatty acid methyl ester	C_19_H_38_O_2_	298	7.12
12	17.87	trans-13-octadecenoic acid	Fatty acid	C_18_H_34_O_2_	282	2.21
13	18.7	7-methyl-Z-tetradecen-1-ol acetate	Ester	C_17_H_32_O_2_	268	13.34
14	24.29	Diisooctyl phthalate	Benzoic acid ester	C_24_H_38_O_4_	390	9.37
15	27.71	Ethyl iso-allocholate	Steroid	C_26_H_44_O_5_	436	4.87
16	30.65	Oleic acid, 3-(octadecyloxy)propyl ester	Fatty alcohol	C_39_H_76_O_3_	592	1.34
17	37.33	Cholest-5-en-3-ol, 24-propylidene-, (3fl)-	Steroid	C_30_H_50_O	426	18.17

**Table 3:** The physicochemical and pharmacokinetic properties of the lipophilic compounds detected in macroalga *T. cylindrica* lipophilic extracts with a high binding affinity toward DM and COVID-19-related protein targets.

Compound	Physiochemical Properties of Compounds Based on the Lipinski Rule				
	Molecular Weight (Dalton)	Hydrogen Bond Donors	Hydrogen Bond Acceptors	Log P	Molar Refractivity (g/mol)
	<500	≤10	<10	≤5	40–130
Compound1	334.45	1	2	4.47	101.09
Compound2	334.45	1	2	6.34	123.61
Compound3	412.69	1	1	6.62	132.75

## Data Availability

The data presented in this study are available on request from the corresponding author. The data are not publicly available due to ethical restrictions.
